# The Conglomerate

**Published:** 1891-11

**Authors:** 


THE CONGLOMERATE: A weekly newspaper published by the patients at the New York State Homœopathic Hospital for the Insane at Middletown, N. Y. Price, one dollar per year; three months, twenty-five cents.

This is a clever little journal of eight pages, full of interesting matter. The number for September 16th contains an excellent article entitled "The Curability of Mental and Nervous Diseases under Homœopathic Medication," by Dr. Selden H. Talcott, Chief Physician and Superintendent of the Hospital. The paper was read at the Homœopathic Medical Congress at Atlantic City, in June last, accompanied by some convincing statistics.

A war is now being waged against the Middletown Asylum by certain correspondents of the New York *Medical Times*, and *The Conglomerate* is making able defense with the aid of the before-mentioned article of Dr. Talcott.

